# Geometry of turbulent dissipation and the Navier–Stokes regularity problem

**DOI:** 10.1038/s41598-021-87774-y

**Published:** 2021-04-23

**Authors:** Janet Rafner, Zoran Grujić, Christian Bach, Jakob Andreas Bærentzen, Bo Gervang, Ruo Jia, Scott Leinweber, Marek Misztal, Jacob Sherson

**Affiliations:** 1 ScienceAtHome, Center for Hybrid Intelligence, Department of Physics and Astronomy, Aarhus University, Aarhus, Denmark; 2 Department of Mathematics, University of Virginia, Charlottesville, VA USA; 3 Department of Computer Graphics, Technical University of Denmark, Lyngby, Denmark; 4 Department of Engineering, Aarhus University, Aarhus, Denmark; 5 Darden School of Business, University of Virginia, Charlottesville, VA USA; 6 Apple INC., San Francisco, CA USA; 7 3Shape TRIOS A/S, Copenhagen, Denmark

**Keywords:** Fluid dynamics, Applied mathematics

## Abstract

The question of whether a singularity can form in an initially regular flow, described by the 3D incompressible Navier–Stokes (NS) equations, is a fundamental problem in mathematical physics. The NS regularity problem is super-critical, i.e., there is a ‘scaling gap’ between what can be established by mathematical analysis and what is needed to rule out a singularity. A recently introduced mathematical framework—based on a suitably defined ‘scale of sparseness’ of the regions of intense vorticity—brought the first scaling reduction of the NS super-criticality since the 1960s. Here, we put this framework to the first numerical test using a spatially highly resolved computational simulation performed near a ‘burst’ of the vorticity magnitude. The results confirm that the scale is well suited to detect the onset of dissipation and provide numerical evidence that ongoing mathematical efforts may succeed in closing the scaling gap.

## Introduction

Humans have been fascinated with the geometry of fluid flows for centuries. Well-known examples of artistic renditions of ‘coherent vortex structures’ appearing in nature include the woodblock print *Great Wave off Kanagawa* by Katsushika Hokusai, Vincent Van Gogh’s *Starry Night* and Leonardo Da Vinci’s drawings of eddy motion. Da Vinci’s studies were in fact among the first scientific studies of turbulent motion which then assumed a more rigorous form in the fundamental works of Kolmogorov^1–3^, Onsager^4,5^, Taylor^6,7^, Weizsacker^8^ and others in the first half of the twentieth century. One of the cornerstones of the turbulence phenomenology is the concept of the energy cascade: there is a nonlinear transfer of energy from larger to smaller scales; once a sufficiently small scale is reached, the diffusion takes over and the energy starts to dissipate in the form of heat. Despite much progress over the decades, a complete, rigorous theory of turbulence remains elusive; in particular, the question as to the role that coherent vortex structures play in the theory of turbulent dissipation.

A flow of 3D incompressible, viscous, Newtonian fluid is described by the 3D Navier–Stokes equations (NSE),

$$\begin{aligned} \frac{\partial \pmb {u}}{\partial t} + (\pmb {u}\cdot \nabla )\pmb {u}=-\nabla p + \nu \triangle \pmb {u} + \pmb {f}, \end{aligned}$$

supplemented with the incompressibility condition $\, \nabla \cdot \, \pmb {u} = 0$, where $\pmb {u}$ is the velocity of the fluid, *p* is the kinematic pressure, $\nu$ is the kinematic viscosity, and $\pmb {f}$ the external force. Taking the curl yields the vorticity formulation,

$$\begin{aligned} \frac{\partial \pmb {\omega }}{\partial t} + (\pmb {u} \cdot \nabla ) \pmb {\omega } = \nu \triangle \pmb {\omega } + (\pmb {\omega } \cdot \nabla ) \pmb {u} + \, \nabla \times \pmb {f} \end{aligned}$$

where $\pmb {\omega }$ is the vorticity of the fluid, $\pmb {\omega } = \, \nabla \times \pmb {u} \,$. The left-hand side represents the transport of the vorticity by the velocity, the first term on the right-hand side generates the diffusion, and the second one is the vortex-stretching term; these are the three principal physical mechanisms in the system. Despite its apparent simplicity, the mathematical theory of the 3D NSE is fundamentally incomplete. In particular, since the pioneering work of Leray in the 1930s^9^, the question of whether a finite-time singularity can form in an initially regular (smooth) unforced flow is still open, and is one of the remaining *Millennium Prize Problems* put forth by the Clay Mathematics Institute (customarily referred to as ‘the NS regularity problem’). Note that—in this context—a temporal point $T^*$ is a *singularity* if the solution is regular on some time-interval $(T^*-\epsilon , T^*)$ and the limit of the maximum of the velocity (or—equivalently—the vorticity) magnitude is infinite as the time variable approaches $T^*$. Such singularity could potentially form in either of the following two cases i) in Eulerian dynamics (zero viscosity), where a formation of ever smaller scales—paired with the formation of ever larger velocity gradients could continue indefinitely or ii) in the viscous case, if the formation of ever smaller scales stays above the threshold of the dissipation scale (shrinking to zero slower than the dissipation scale, as the flow approaches the singularity).

The NS regularity problem is *supercritical*; in other words there is a ‘scaling gap’ between any known regularity criterion and the corresponding *a priori* bound. Here a ‘regularity criterion’ refers to an analytic or geometric property of the solution sufficient to rule out a singularity, while an ‘*a priori* bound’ refers to an analytic or geometric property of the solution that can be rigorously derived from the NSE. Moreover, since the fundamental independent works of Ladyzhenskaya, Prodi and Serrin, as well as Kato and Fujita, in the 1960s^10–13^, no one has improved upon the regularity criteria, with respect to the intrinsic NS scaling transformation, and all the *a priori* bounds have been on the scaling-level of Leray’s original energy bound. Recently, however, a new mathematical framework^14^ enabled the first *scaling reduction* of the NS super-criticality (more precisely, the *a priori* bound derived represents a $40\%$ improvement over the energy level bounds) since the 1960s; the key concept in the theory is a suitably defined ‘scale of sparseness’ (a precise definition resides in the following section) of the super-level sets of the positive and the negative parts of the vorticity components. At this point, a natural question arises of whether there might be an intrinsic obstruction to advancing this method, or whether this new framework might have more to offer (a further reduction of the scaling gap).

In this work we present the first numerical analysis of this novel geometric scale applied to two conventional scenarios (1) a Kida-vortex initialized simulation: a model flow for the study of possible singular events in the 3D incompressible fluid flows (away from the boundary), in particular because it exhibits a ‘burst’ of vorticity maximum, resembling a finite singularity^15^ (2) a simulation in the realm of fully developed, homogeneous, isotropic turbulence. One should note that homogenous and isotropic refers to the velocity description; the vorticity description is inherently inhomogeneous and anisotropic. The main result of this paper demonstrated that in the former case the scale of sparseness is not only actualized well beyond the guaranteed a priori bound^14^, but also just beyond the critical bound^14^ sufficient for the diffusion to fully engage (overpowering the nonlinearity) and prevent the further growth of the vorticity magnitude. This result provides numerical confirmation that the aforementioned mathematical framework is capable of accurately detecting the onset of turbulent dissipation, and furnishes a necessary validation of the current theoretical efforts in closing the scaling gap in the NS regularity problem within the framework. In addition, we provide arguments that shed new light on some of the classical work in the computational simulations of *intermittent events* in turbulence. More specifically, based on the theory presented in^14^ we provide–for the first time—a rigorous explanation for the eventual ‘slumps’ in the ‘bursts’ of the vorticity magnitude observed in computational modeling of the Kida vortex-initialized flows^15–17^.

In the second scenario, we analyze data sampled from the forced isotropic turbulence simulation publicly available from the Johns Hopkins Turbulence Data Base (JHTDB). This data set had previously only been analyzed for spectral scales, and this work presents the first analysis of the geometric properties of the simulated flow. Since the scale of sparseness is a *small scale* (e.g., in the case of an ensemble of filamentary ‘regions of intense vorticity’ (RIVs), it is comparable to the transversal scale, i.e., to the diameter of an RIV, and not to the longitudinal scale, i.e., to the length of an RIV), we expect it to exhibit a scaling trend consistent with being in the dissipation range. This was indeed confirmed by performing time series analysis on the data sampled from JHTDB. The results are presented in the "Appendix".

Finally, as we believe the general concept of the ‘scale of sparseness’ could be potentially impactful in many other situations in science and engineering, it is meaningful to take a closer look at how to optimize the data analysis algorithms. In the absence of heuristics, all RIVs must be investigated and an exhaustive search for the maximum *r* value conducted (*r* denotes a scale comparable to the scale of sparseness). This brute force approach is computationally demanding. One field that seeks to systematize the search for heuristics is Computational Citizen science. Computational Citizen science is based on a research problem with a complete mathematical description and a unique and easily calculable score/quality for each user solution (easy validation), which enables a direct comparison between algorithmic performance and the human computation. This type of citizen science lends itself to highly complex (multi-dimensional) natural science research problems.

Computational Citizen science has proven a useful technique for optimization in other fields such as protein folding^18^, RNA mapping^19^ and quantum physics^20,21^. Thus, we explored the use of a digital, gamified interface (seen in Fig. 2) which allowed 700 citizen-scientists from around the world to participate in the study of the scale of sparseness. In the context of the Kida vortex-initialized simulation, quantitative analysis of players’ searches shows that they were able to achieve a comparable level of accuracy in identifying the $r_{max}$ (which was visualized as the radius of the largest sphere that can fit inside each RIV; a scale comparable to the scale of sparseness). The results can be seen in Fig. 3).

The paper is organized as follows. "Mathematical background" section provides a mathematical background necessary to present our results (in particular, the mathematical framework suitable for quantifying the phenomenon of ‘spatial intermittency’ introduced in^14^), "Results" section exposes the main results, "Discussion" section contains the discussion and the outlook, and "Methods" section delineates the methods utilized.

## Mathematical background

Let us start by recalling several concepts from mathematical analysis. Let $\Omega$ be a subset of ${\mathbb {R}}^3$. Recall that a function $\pmb {f} : \Omega \rightarrow {\mathbb {R}}^3$ is Lipschitz-continuous if there exists a constant *L* such that $\Vert \pmb {f}(x)-\pmb {f}(y)\Vert \le L \Vert \pmb {x}-\pmb {y}\Vert$ for all $\pmb {x}$ and $\pmb {y}$ in $\Omega$. Complementary, $\pmb {f}$ is Hölder-continuous with the exponent $\alpha$, $0< \alpha < 1$, if there exists a constant *A* such that $\Vert \pmb {f}(x)-\pmb {f}(y)\Vert \le A \Vert \pmb {x}-\pmb {y}\Vert ^\alpha$ for all $\pmb {x}$ and $\pmb {y}$ in $\Omega$. Let *f* be a Lebesgue measurable, scalar or vector valued function on an open set $\Omega$ in the Euclidean space. Recall that for an exponent *p*, $1 \le p < \infty$, the Lebesgue space of *p*-integrable functions on $\Omega$, denoted by $L^p$, is determined by finiteness of the $L^p$-norm,

$$\begin{aligned} \Vert f\Vert _p = \biggl ( \int _\Omega |f(x)|^p \, dx \biggr )^\frac{1}{p}. \end{aligned}$$

In the limit case $p=\infty$, the corresponding space of essentially bounded functions, $L^\infty$, is determined by finiteness of the $L^\infty$-norm,

$$\begin{aligned} \Vert f\Vert _\infty = \sup _{x \in \Omega } |f(x)| \end{aligned}$$

(the supremum is taken almost everywhere-*x* with respect to the Lebesgue measure). Closely related are the ‘weak Lebesgue spaces’ $L^p_w$ determined by the rate of decay of the volume of the super-level sets of a function. Let $\lambda > 0$ and consider the super-level set of the function *f* cut at the level $\lambda$,

$$\begin{aligned} S_\lambda = \{ x \in \Omega : \, |f(x)| > \lambda \}. \end{aligned}$$

The function *f* belongs to $L^p_w$ if there exists a positive constant *c* such that

$$\begin{aligned} m^3 \biggl (S_\lambda \biggr ) \le \frac{c^p}{\lambda ^p} \end{aligned}$$

for all $\lambda > 0$ ($m^3$ denotes the 3-dimensional Lebesgue measure). An $L^p$ function is automatically in $L^p_w$; the converse is false.

A key role played by geometry of the flow had been announced at least as far back as G. I. Taylor’s fundamental paper *Production and dissipation of vorticity in a turbulent fluid* in 1930s. Taylor inferred his thoughts on turbulent dissipation partly from the wind tunnel measurements of a turbulent flow past a uniform grid, concluding the paper with the following statement: “It seems that the stretching of vortex filaments must be regarded as the principal mechanical cause of the high rate of dissipation which is associated with turbulent motion”^7^.

A pioneering event in incorporating geometry of the flow in the mathematical study of possible singularity formation in the 3D NSE took place in P. Constantin’s paper *Geometric statistics in turbulence*^22^. This approach was based on a singular integral representation for the stretching factor in the evolution of the vorticity magnitude, and the key observation was that the kernel had a geometric flavor; more precisely, it was *depleted* by the *local coherence* of the *vorticity direction* (this has been referred to as ‘geometric depletion of the nonlinearity’). The first quantification of this phenomenon appeared in^23^, revealing that postulating the Lipschitz-continuity of the vorticity direction in the regions of intense vorticity suffices to guarantee that the flow initiated at a regular initial configuration does not develop a singularity.

Subsequent results include^24^ where it was shown that the assumption on the Lipschitz-continuity can be scaled down to $\frac{1}{2}$-Hölder-continuity^25^, in which a complete *spatiotemporal localization* of the $\frac{1}{2}$-Hölder-continuity regularity criterion was performed, and^26^ in which the authors presented a two-parameter family of local, hybrid geometric-analytic, *scaling-invariant* regularity criteria, based on a *dynamic balance* between the coherence of the vorticity direction and the vorticity magnitude.

Another (in addition to the local coherence of the vorticity direction) morphological signature of the regions of intense vorticity is *spatial intermittency*—this has been well-documented in computational simulations as well as experiments. Classical references on the morphology of turbulent flows include^27–31^. One should also note that—in contrast to the more traditional approach of chasing the super-high Reynolds number regimes—a relatively recent work^32^ revealed that, in sufficiently resolved flows, the universality in the realm of the small-scale structures already transpires at low to moderate Reynolds numbers.

The concepts of local 1D and 3D ‘sparseness’ introduced in^33^ were designed to model the spatial intermittency in a way amenable to rigorous mathematical analysis based on the 3D NSE. Henceforth, sparseness will refer to 3D sparseness defined below.

Let *S* be an open subset of ${\mathbb {R}}^3$, $\delta \in (0,1)$, $\pmb {x}_0 \in {\mathbb {R}}^3$, and $r \in (0, \infty )$. *S* is *3D* $\delta$*-sparse around* $\pmb {x}_0$ *at scale* *r* if

$$\begin{aligned} \frac{m^3\bigl (S\cap B(\pmb {x}_0,r)\bigr )}{m^3\bigl (B(\pmb {x}_0,r)\bigr )} \le \delta . \end{aligned}$$

The main idea presented in^33^ was to utilize sparseness of the vorticity super-level sets via the *harmonic measure maximum principle* (for the essentials on the harmonic measure in the complex plane see, e.g.,^34^); in short, the sparseness translates into smallness of the harmonic measure associated with the regions of the intense vorticity which—in turn—provides a bound on the $L^\infty$-norm, controlling a possible finite time blow-up. A key PDE technique utilized here was deriving the lower bounds on the radius of spatial analyticity of the solution in the $L^\infty$-type spaces. The principal result obtained in^33^ states that as long as the suitably defined vorticity super-level sets are sparse at the scale comparable to the radius of spatial analyticity measured in $L^\infty$, no finite time blow-up can occur. Since the local-in-time spatially analytic smoothing is simply a (strong) manifestation of the diffusion, this result is consistent with the physics of turbulent dissipation.

As noted in the Introduction, the Navier–Stokes regularity problem is *supercritical*, i.e., there is a ‘scaling gap between any known regularity criterion and the corresponding a priori bound (with respect to the intrinsic NS scaling). On one hand, since the fundamental (independent) works of Ladyzhenskaya, Prodi and Serrin, as well as Kato and Fujita, all in 1960s, all the regularity classes have been (at best) scaling-invariant, and on the other hand, all the a priori bounds have been at the energy level. This is exemplified in the scale of uniform-in-time, $L^p$-in-space Banach spaces as follows: the regularity class is $L^\infty ((0,T), L^3)$^35^, while corresponding *a priori* bound is $L^\infty ((0,T), L^2)$. A more comprehensive summary of the scaling gaps can be found in^36^.

**Figure 1:**
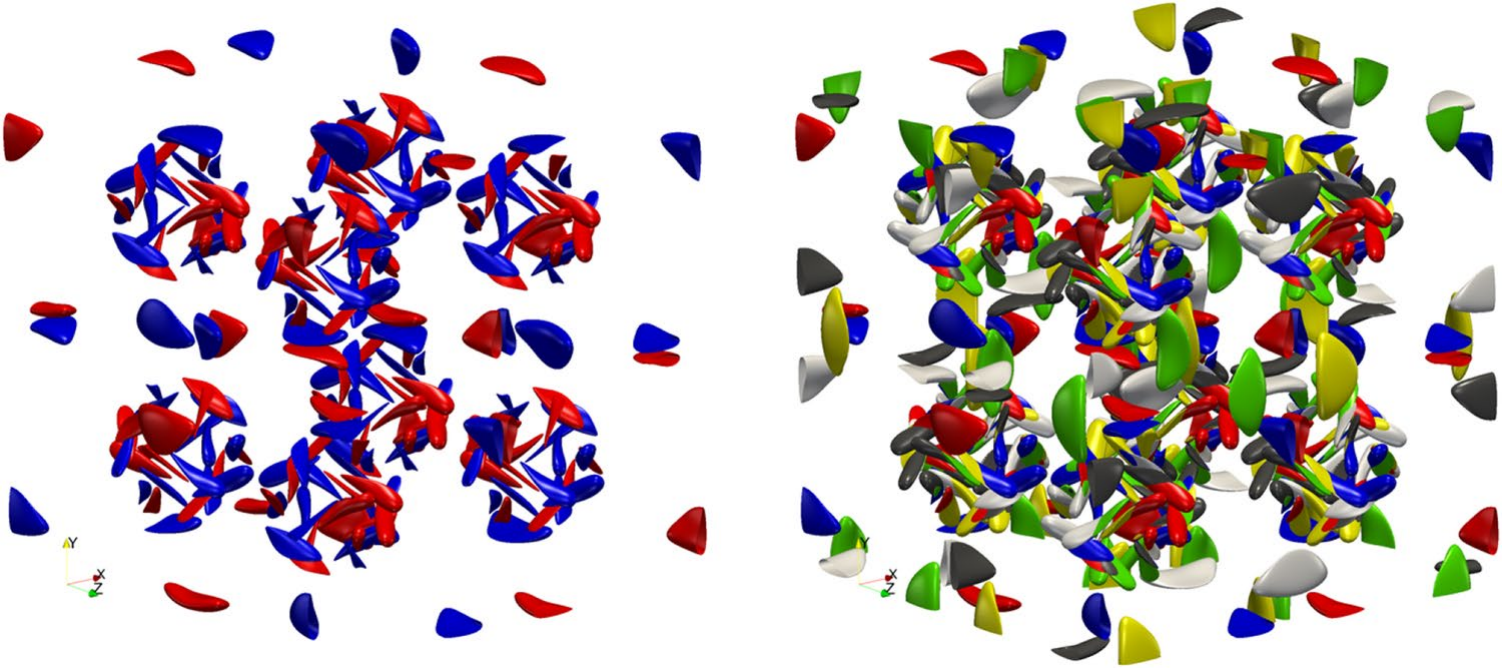
An illustration of the difference between sparseness of the super-level sets of the individual components and the full vectorial super-level set. The figure on the left depicts the super-level sets of the positive and the negative parts of the first component of the vorticity field, while the figure on the right—the union of the six individual super-level sets—corresponds to the super-level set of the vorticity magnitude. Obtained from the Kida simulation.

It turned out that redesigning the theory introduced in^33^ around the super-level sets of the *vorticity components* instead of the vectorial super-level sets (see Fig. 1) produced the first ‘modern era’ *algebraic reduction* of the scaling gap–since 1960s, all the improvements were logarithmic in nature, regardless of the mathematical framework utilized.

**Figure 2:**
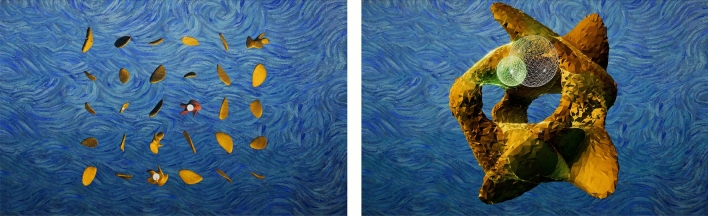
Screen captures from the Turbulence computational citizen science game showing first the selection of the RIVs and second, moving a sphere inside an individual RIV. Recall, the radius of the largest sphere that can fit inside each individual RIV represents the scale of sparseness.

Details can be found in^14^; here we present the classes $Z_\alpha$ as well as the synopsis of the two main results encoded in the $Z_\alpha$-formalism. Henceforth, for a vector field $\pmb {f}$, the positive and the opposite of the negative parts of the components $f_i$ will be denoted by $f_i^\pm$, $i=1, 2, 3$.

For a positive exponent $\alpha$, and a selection of parameters $\lambda \in (0,1)$, $\delta \in (0,1)$ and $c_0>0$, the class of functions $Z_\alpha (\lambda , \delta ; c_0)$ consists of bounded, continuous functions $\pmb {f} : {\mathbb {R}}^3 \rightarrow {\mathbb {R}}^3$ subjected to the following uniformly local condition. For $\pmb {x}_0 \in {\mathbb {R}}^3$, select the/a component $f_i^\pm$ such that $f_i^\pm (\pmb {x}_0) = \Vert \pmb {f}(\pmb {x}_0)\Vert$ (here, the norm of a vector $\pmb {v}=(a, b, c)$, $\Vert \pmb {v}\Vert$, will be computed as $\max \{|a|, |b|, |c|\}$), and require that the super-level set

$$\begin{aligned} \biggl \{ \pmb {x} \in {\mathbb {R}}^3: \, f_i^\pm (x) > \lambda \Vert \pmb {f}\Vert _\infty \biggr \} \end{aligned}$$

be $\delta$-sparse around $\pmb {x}_0$ at scale $\frac{1}{c} \frac{1}{\Vert \pmb {f}\Vert _\infty ^\alpha }$, for some *c* comparable (with respect to the order of magnitude) with $c_0$. Enforce this for all $\pmb {x}_0 \in {\mathbb {R}}^3$. (In short, we require sparseness of the/a *locally maximal* component only.)

In this setting, the regularity class is $Z_\frac{1}{2}$ (pointwise-in-time, in the context of a blow-up argument)^14^; this is on the scaling level of all the classical regularity criteria. The a priori bound obtained lives in $Z_\frac{2}{5}$ (pointwise-in-time, in the context of a blow-up argument)^14^; since the energy level class is $Z_\frac{1}{3}$, this represents a $40\%$ reduction of the scaling gap in the $Z_\alpha$ framework!

Let us note that since the focus of this study, and more broadly this approach/framework, is on the possibility of the spontaneous formation of singularities in the NS system, we are considering the solutions that are smooth (and in fact spatially analytic) up to a possible singular time. If one is interested in the weak solutions–either to the NS or to the Euler system–as the primary object, then the list of the critical exponents of interest grows. In particular, the exponents identifying the uniqueness classes, as well as the classes that are amenable to the application of the *h*-principle emerge (for a discussion in the context of the Euler system, see^37^). Here, since we are in the setting of the smooth solutions, the main critical parameters of interest are the ones identifying the regularity class, the scaling-invariant class, the class featuring the best/strongest *a priori* bound, and the energy level class. In the $Z_\alpha$ scale, $\alpha$-values delineating these classes are $\frac{1}{2}, \frac{1}{2}, \frac{2}{5}$, and $\frac{1}{3}$, respectively, $\frac{1}{3}< \frac{2}{5} < \frac{1}{2} = \frac{1}{2}$.

It is instructive to make a quick comparison between the $Z_\alpha$-classes and the weak Lebesgue classes $L^p_w$ determined solely by the rate of decay of the volume of the super-level sets, encoding no geometric information. On one hand, it is straightforward that $\displaystyle { f \in L^p_w \ \text{ implies } \, f \in Z_\alpha \ \text{ for } \ \alpha =\frac{p}{3}}$ (for a given selection of $\lambda$ and $\delta$, the size parameter $c_0$ will depend on the $L^p_w$-semi-norm of *f*); on the other hand, in the geometrically worst case scenario–the whole super-level set being clumped into a single ball–being in $Z_\alpha$ is consistent with being in $L^{3 \alpha }_w$ (however, one should note that–in general—membership in $Z_\alpha$ does not impose any decay on the volume of the super-level sets since it provides information on the suitably defined scale of the ‘largest piece’ but no information on the number of ‘pieces’). This observation reveals that a ‘geometrically blind’ scaling counterpart of the *a priori* bound in $Z_\frac{2}{5}$ would be the bound $\omega \in L^\infty ((0,T), L^\frac{6}{5}_w)$ which is well beyond state-of-the-art ($\omega \in L^\infty ((0,T), L^1)$^38^), demonstrating a clear advantage of working within the $Z_\alpha$ realm compared to the functional classes traditionally utilized in the study of the 3D NS regularity problem.

## Results

### Kida-vortex initialized flow: towards closing of the scaling gap

Recall that in the $Z_\alpha$ framework, the rigorous regularity and *a priori*-bound classes are $Z_\frac{1}{2}$ and $Z_\frac{2}{5}$, respectively^14^. In order to test whether there may be an obstruction to advancing the $Z_\alpha$ a priori bounds even further—with the ultimate goal of *closing* the scaling gap—we considered a Kida-vortex initialized flow, a flow with the highly symmetric initial condition exhibiting a sharp increase (a ‘burst’) of the vorticity maximum ‘simulating’ a finite-time singularity^15–17^.

Attention was focused on a time interval leading to the peak of $\Vert \pmb {\omega }(t)\Vert _\infty$, and the aim was to investigate a possibility of a power-law dependence between the actual geometric scale of sparseness *r*(*t*) and the diffusion scale $d(t)=\frac{\nu ^\frac{1}{2}}{\Vert \pmb {\omega }(t)\Vert _\infty ^\frac{1}{2}}$ of the form $r \sim d^\alpha$ (recall that *d* is a lower bound on the radius of spatial analyticity; the importance of this scale was identified and rigorously established in^39^). A presence of a power-law scaling would indicate that the scale of sparseness *r* is a *bona fide* small scale in the sense of turbulence phenomenology. In addition–since both *d* and *r* are valued between 0 and 1 (empirically, throughout the data set)–detecting a power law with an exponent $\alpha > 1$ would mean that the solution in view is entering the diffusion regime (as depicted in the $Z_\alpha$ framework) and is, in fact, contained in the regularity class $Z_\frac{1}{2}$.

**Figure 3:**
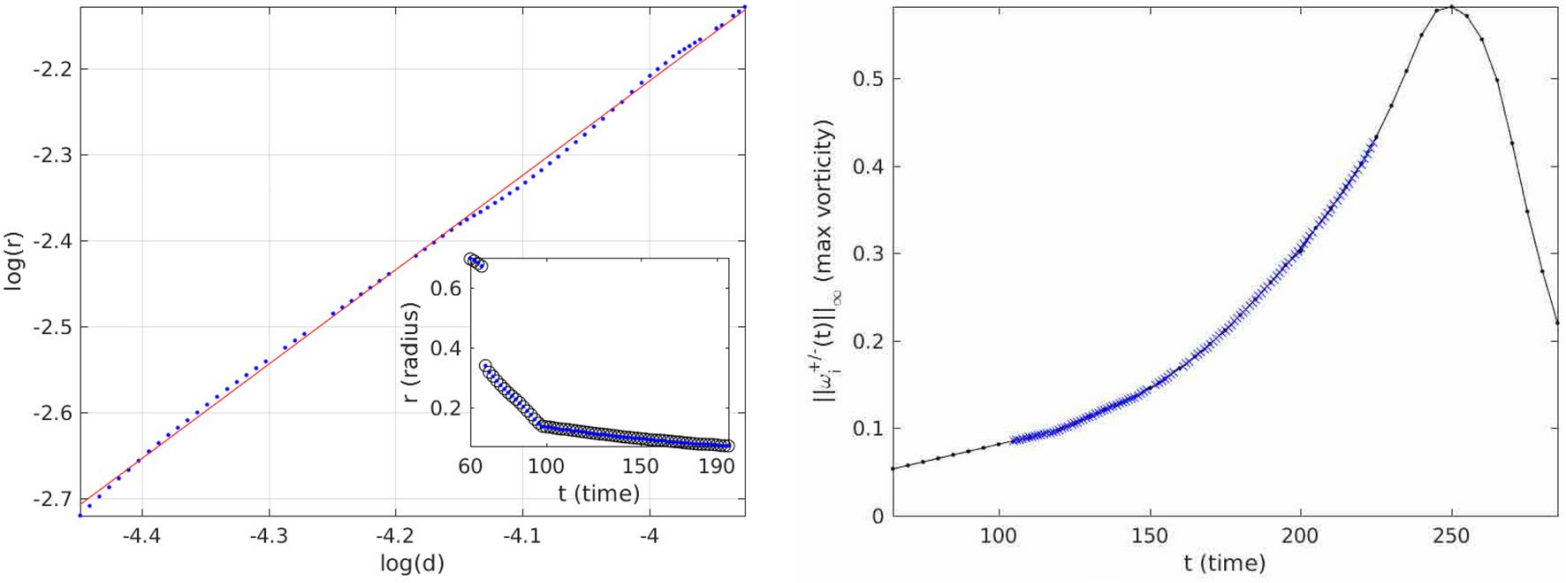
The figure on the left is a Log–log plot of the scale of three dimensional sparseness (of the vorticity components) *vs.* the diffusion scale. The summary of the regression is as follows: fit = $[1.098 \pm 0.009] \log d + [2.17 \pm 0.04]$. The inset compares the algorithmically calculated $r_{max}$ [the small dots] to the $r_{max}$ [the small circles] found by citizen scientists. The figure on the right is the time-evolution of the vorticity magnitude. The highlighted region indicates where the data was sourced from.

Indeed, data analysis of the time-interval of interest-sourced from a spatially highly-resolved simulation (for some details see the Methods)—revealed a very strong evidence of a power-law scaling; moreover, the scaling exponent $\alpha$ crystalized at $1.098 \pm 0.009$ (cf. Fig. 3). This was very exciting to see.

### Kida-vortex initialized flow: a rigorous explanation of the Boratav–Pelz computational results

Boratav and Pelz considered the Kida vortex flows as—among other things—a laboratory for the computational study of the possible singularity formation in solutions to the 3D NSE and Euler flows. In particular, they discovered a time-interval of extreme intermittency (preceding the peak of the vorticity maximum) in which the local pointwise and integrated quantities increase sharply, noting that “The increase is so sudden and sharp that questions arise as to whether some of these quantities would diverge in finite time or not”^15^.

They performed a singularity analysis revealing that the vorticity maximum–in a time-interval leading to its peak–scales closely to the self-similar, critical scaling of $\frac{1}{t-T^*}$. This could, in principle, be an indication of the build-up of a scaling-invariant singularity. Nevertheless, the simulations consistently showed an eventual disruption in the (approximately) self-similar, critical scaling, a formation of the peak, and a subsequent dissipation of the flow, prompting them to conclude that “However, the increase in peak vorticity stops at a certain time, possibly due to viscous dissipation effects”^15^.

As noted in the previous subsection, the analysis of the data sourced from an interval leading to the peak of the vorticity maximum within the $Z_\alpha$ framework demonstrated that the flow entered a regime beyond the critical diffusion class $Z_\frac{1}{2}$. In other words, sparseness of the super-level sets of the vorticity components was sufficient for the harmonic measure maximum principle to engage and prevent a further growth of the vorticity maximum, providing a rigorous justification of the observed ‘slump’ and dissipation. Nevertheless, it is worth noticing that the exponent observed in our analysis ($1.098 \pm 0.009$) is only slightly larger than the critical exponent (1), confirming a close proximity to the criticality.

## Discussion

The main goal of this paper was to investigate whether there was an intrinsic obstruction to further reduction of the scaling gap in the 3D Navier–Stokes regularity problem, within the mathematical framework based on the suitably defined scale of sparseness of the regions of intense fluid activity. The idea was to perform a spatially highly resolved computational simulation of a Kida vortex-initialized flow, and investigate the pertinent scaling properties as the flow approached a peak of the vorticity magnitude. The data analysis exhibited that—in a time-interval leading to the burst–the scaling signature of the scale of sparseness was slightly beyond the critical scaling (the scaling exponent of $1.098 \pm 0.009$ *vs.* 1), setting the stage–within the aforementioned framework—for the diffusion to fully engage and prevent further growth of the vorticity magnitude (shortly, no roadblocks to criticality were detected). To put this finding in perspective, on one hand, a scaling exponent less than 1 would indicate that the new geometric framework does not offer a good gauge of whether the flow has entered a diffusion regime. On the other hand, a scaling exponent significantly larger than one (i.e., significantly away from the critical scaling) would not be consistent with the previously observed near-criticality features of the Kida bursts (cf.^15^).

The new geometric framework was designed to study the geometry of turbulent dissipation in the vicinity of a hypothetical singularity (or a burst of the vorticity magnitude), regardless of the initial configuration. The Kida vortex-initialized flow was chosen as a notable example of a flow capable of producing a sudden burst. It would be interesting to perform a similar analysis of other flows with this capability (e.g., the flow studied in^40^, featuring several peaks of the vorticity magnitude, also initialized at an initial configuration concentrated on the first couple of Fourier modes, but with no imposed symmetries).

The exploratory results from the computational citizen science game give first indications that it may be beneficial to study how an initial screening of human ’spot sorting’ of these RIVs could potentially speed up our algorithmic processes. This approach could be particularly useful when analyzing vast amounts of structures with comparable bounding box volumes, as this is the condition for reducing the number of RIVs analyzed with a distance field calculation. Apart from potential implications for analysis in computational fluid dynamics, this could offer significant insights within neurocomputation^41^ and computational geometry^42^ while at the same time being an excellent outreach initiative to engage the general public in natural science research.

The results exposed in this paper have provided a significant boost to the continuation of rigorous analysis, based on a countable hierarchy of function classes. Shortly, the original framework^14^ was based on sparseness of the super-level sets of the vorticity, which could be viewed as the super-level sets of the first-order spatial fluctuation of the velocity. It turns out that considering the super-level sets of the higher-order spatial fluctuations of the velocity field yields a further reduction of the scaling gap. This is an ongoing work, and will be reported elsewhere.

In conclusion, the numerical work presented here represents the first application of the abstract mathematical framework based on the concept of the ‘scale of sparseness’ to concrete flows and our numerical success provides strong encouragement that this mathematical framework may provide novel insights in many realms of fluid dynamics in both science and engineering.

## Methods

The Kida vortex initial configurations were introduced in^43^. The class features a high number of symmetries, all preserved by the Navier–Stokes flows. The symmetries include periodicity, bilateral symmetry, rotational symmetry and permutational symmetry of the velocity components. More specifically, we considered the following one-parameter family of the initial conditions,

$$\begin{aligned} u_{0,x}&= \sin x \bigl ( \cos (3y) \cos z - \cos y \cos (3z)\bigr ) U_0\\ u_{0,y}&= \sin y \bigl ( \cos (3z) \cos x - \cos z \cos (3x)\bigr ) U_0\\ u_{0,z}&= \sin z \bigl ( \cos (3x) \cos y - \cos x \cos (3y)\bigr ) U_0 \end{aligned}$$

(cf.^44^). The Reynolds number components were chosen as follows, $U_0 = 0.01, L = 2 \pi$ ($2 \pi$-periodic box), $\nu = \frac{1}{3} 10^{-4}$, resulting in the Reynolds number $Re = \frac{U_0 L}{\nu }$ of approximately 2000.

One should note that the scale of sparseness is sensitive to the parameters $\lambda$ and $\delta$ introduced in the definition of the $Z_\alpha$ classes in Section II (the remaining parameter $c_0$ is simply the size parameter). Since the goal of the simulation was to compare the actual scale of sparseness in the flow *r* with the rigorous lower bound on the dissipation scale *d* derived in^14^, we kept the values consistent with the ones used in^14^. More precisely, $\lambda$ was set to $\frac{1}{2}$ (the value utilized in^14^ was $\frac{1}{2M}$ where *M* was slightly larger than 1), and the permitted range for $\delta$ was $\bigl [\frac{1}{2}, \frac{7}{8}\big ]$ (the value utilized in^14^ was $\frac{3}{4}$).

The data set was created utilizing a mixed spectral finite elements-Fourier method on a ${1024}^3$ spatial grid. The *XY*-plane was discretized with a Galerkin pseudospectral discretization and extended in the z-direction with a Fourier discretization. This allows for a scalable solver and also exploits the speed of the Fast Fourier Transform similarly as in^45^. The Navier–Stokes equation was solved using an operator splitting method (Velocity Correction Scheme) leading to several systems of equations of reduced complexity to be solved instead of one large matrix system. That is while maintaining a splitting error on the order of the overall numerical discretization error. The temporal discretization was done with Implicit-explicit (IMEX) schemes. Among other schemes, we specifically used an IMEX 3rd order scheme, which uses a third order Adams–Bashforth scheme for the convective term and a third order Adams–Moulton for the Diffusion term—The first order IMEX scheme analogously uses a combination of Euler schemes. While the symmetries were not fully utilized throughout the code, they were fully utilized in reducing the computational complexity associated with estimating the scale of sparseness corresponding to different components of the vorticity field.

The data was analyzed mainly using a conventional algorithmic approach but also an exploratory citizen science approach described in the outlook. The algorithmic approach used a signed distance field calculation^42^ where distances were computed from triangle mesh representations of the RIVs. A distance field for a surface is a mapping, $d: {\mathbb {R}}^3 \rightarrow {\mathbb {R}}$, from a point in space, ${\mathbf {p}}$, to the distance from ${\mathbf {p}}$ to its closest point on the surface. The sign of the distance field allows us to tell inside from outside, and the largest interior distance is also the radius of the largest sphere fully contained in the RIV. To compute the distance efficiently at a given point, ${\mathbf {p}}$, a bounding box hierarchy was used to find the triangles most likely to contain the point at a closest distance to $\mathbf{p}$. To compute the sign of the distance, we used ray casting to perform multiple tests for each point in order to determine if ${\mathbf {p}}$ were inside or outside the surface. On the other hand, significant optimizations were possible due to the fact that, for a given time slice, once an estimate of the greatest radius, *r*, had been found, we only needed to consider other RIVs if their bounding boxes were large enough to contain a sphere of radius *r*. Furthermore, we optimized the search for the largest radius within a given RIV by recursively computing distances at finer grid resolutions in the vicinity of the largest distance value found at the next coarser resolution.

## Figures and Tables

**Figure 4:**
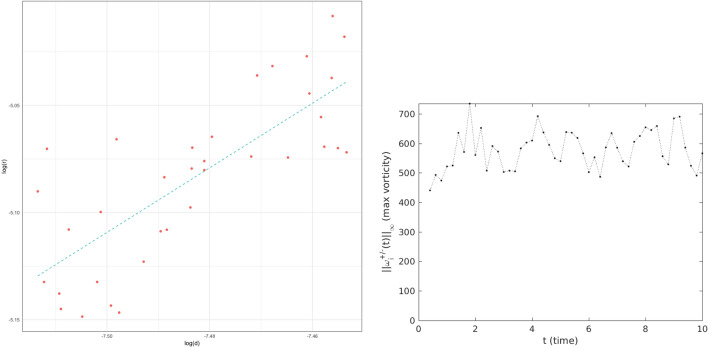
The figure on the left represents the linear regression after the cyclic component of the data was filtered out and the figure on the right the time-evolution of the vorticity magnitude. The summary of the regression is as follows fit = $[1.5 \pm 0.2] \log d + [6.1 \pm 0.16]$.

## Appendix: Homogeneous, isotropic turbulence

Here, we consider a forced isotropic turbulence simulation made available through the JHTDB. The flow is kept in the statistical stationary state of the fully developed turbulence by perpetually injecting the energy at large scales (at low Fourier modes, effectively), keeping the kinetic energy constant within the Fourier modes of the length less or equal to two. Any stationarity is exhibited in the average only, and any pointwise-in-time-measured extremal quantities (e.g., the vorticity maximum, *d* and *r*) are expected to exhibit significant fluctuations.

As already noted in Introduction, the scale of sparseness is a small scale and—as such—is expected to trend as a dissipation range quantity. In particular, in the framework of the $Z_\alpha$-classes, we expect it to stay within the confinements of the critical diffusion/dissipation class $Z_\frac{1}{2}$. This was indeed confirmed via time series analysis of the data sampled from the JHTDB. More precisely, before filtering out the cyclic component, the scaling factor was $1.7 \pm 0.6$, and after the filtering $1.5 \pm 0.2$ (cf. Fig. 4). Recall that a scaling exponent greater than one corresponds to the dissipation range.
